# Application of Time Dependent Probabilistic Collision State Checkers in Highly Dynamic Environments

**DOI:** 10.1371/journal.pone.0119930

**Published:** 2015-03-23

**Authors:** Javier Hernández-Aceituno, Leopoldo Acosta, José D. Piñeiro

**Affiliations:** Departamento de Ingeniería Informática y de Sistemas, Universidad de La Laguna, La Laguna, Canary Islands, Spain; Southwest University, CHINA

## Abstract

When computing the trajectory of an autonomous vehicle, inevitable collision states must be avoided at all costs, so no harm comes to the device or pedestrians around it. In dynamic environments, considering collisions as binary events is risky and inefficient, as the future position of moving obstacles is unknown. We introduce a time-dependent probabilistic collision state checker system, which traces a safe route with a minimum collision probability for a robot. We apply a sequential Bayesian model to calculate approximate predictions of the movement patterns of the obstacles, and define a time-dependent variation of the Dijkstra algorithm to compute statistically safe trajectories through a crowded area. We prove the efficiency of our methods through experimentation, using a self-guided robotic device.

## Introduction

In order for an autonomous vehicle to properly operate in an uncontrolled environment, safe trajectories must be ensured so it does not harm itself or pedestrians nearby. This is not a simple calculation, as decisions must be taken in real time to avoid accidents, while still trying to reach a target position as efficiently as possible.

The concept of inevitable collision state (ICS) [1] is defined as the configuration of an autonomous robotic vehicle for which the event of a collision with an obstacle is unavoidable. This definition is valid for environments with objects that remain static or that move following deterministic trajectories. However, as [2, 3] recognize, this is not a safe choice when these obstacles are, for instance, pedestrians.

This situation has been approached using Braking ICS [4], Rapidly-exploring Random Trees [5], Chance Constraints [6], Probabilistic Collision States [7, 8], or combinations thereof [9]. The Probabilistic Collision States method (PCS) assumes probable future occupation areas for each observed object, which depend on the geometry of both the object and the robot.

When first introducing PCS, Althoff *et al.* [8] considered two kind of objects: passive, when they completely ignored the situation of the approaching robot, and active, when they altered their trajectory in order to avoid collisions. All these objects were modeled to move following a uniformly accelerated motion, where the acceleration was not necessarily parallel to the initial velocity and was proportional to the effort the obstacle put into avoiding the robot. A passive object therefore moved with a uniform speed. These parameters were predicted using a random motion model, defined in [10, 11], following a normal distribution, as shown in [12] and its follow-up publication [13].

In order to avoid considering infinite time spans, Althoff *et al.* restricted the movement of the robot to a uniformly decelerated motion, so the acceleration direction was constant relative to the velocity direction, and always forming such an angle that produced a deceleration (their scalar product had to always be negative). This model was insufficient for a real life application, for it was restricted to braking motions and all objects were assumed to move uniformly. Furthermore, the consideration of obstacles actively avoiding a collision could be a dangerous assumption, since a robot should not expect an environment that varies favorably.

Althoff *et al.* also treated all possible locations without considering the evolution in time. Since the position of the obstacles varied during the procedure, the resulting trajectory would be faulty. This problem has been solved before by more computationally expensive planners [14–17].

To offer a safer and more efficient alternative to this model, in this paper we present a Bayesian time-dependent modification of the PCS system, which corrects all these flaws and meets the computational requirements to be implemented in a real autonomous vehicle. Our method relies on obstacle movement prediction and fast trajectory computing, and as such bears conceptual similarities to previous works [18–21]. These works, however, were presented as purely theoretical exercises, whereas our approach is backed up by experimental data obtained from tests on a real environment.

The following section of this paper defines the theoretical models and assumptions on which our calculations are based; we introduce how a collision probability matrix is calculated for a working area during a definite time window; afterwards, we explain how trajectories across this matrix are computed. Our proposal is put to the test in crowded areas, both simulated and real.

## Methods

Our study defines the following basic elements:

Both the vehicle and the obstacles are assumed to exist within a working space *W*, defined as a two-dimensional grid. The area occupied by the robot is named *A*_*R*_ ⊂ *W*, whereas each object *i* takes an area of *A*_*i*_ ⊂ *W*; the total number of obstacles will be denoted as *N*. For the sake of simplicity and more efficient calculations, all areas are considered circular and defined by radii *r*_*R*_ and *r*_*i*_. For more complex shapes, these radii correspond to the circles in which the objects can be inscribed. In all the experiments in this paper, unless stated, *W* is a two-dimensional grid of 50×50 cells.

It is assumed that each object is observed during a certain time span before its future behavior can be modeled. We therefore consider an observed position vector $x_{0\cdots t}^{i}$ for each obstacle *i*. The time interval between two observations is assumed to be as short as possible, and equal to or greater than the processing time required to execute a complete iteration of our algorithm.

Unlike previous works [8], we do not consider unbounded time spans, but definite overlapping time windows that allow the probable location of each object to be calculated for a certain period, similarly to [22, 23]. The length of these time windows, named *T*, can be selected to best suit the needs of each real case. The position of the observed obstacles is estimated for up to *T* time steps into the future. The present time step is named *t*_*P*_; the last time step for which predictions are made is therefore *t*_*T*_ = *t*_*P*_+*T*.

The following subsection of this paper explains how to calculate a matrix of probable locations over the working area *W* for each obstacle, defined by its motion vector $x_{0\cdots t{\;}_{P}}^{i}$, for the chosen time window of size *T*. The global collision matrix *M*, which the robot must consider to properly design a trajectory, must take into account the added probable positions of every observed obstacle. A trajectory of the robot is named *u*(*t*) and is obtained by finding the optimal path through *W* with a minimum collision probability.

### Probabilistic collision matrix

The probable location matrix $m_{t}^{i}$ of an individual object *i* at time *t* may follow any probability distribution required by each particular problem, but typically uses a Gaussian distribution, discretized into a grid model. In order to predict the future positions of the observed obstacles, we apply a sequential Bayesian model, which combines perceived location and previously calculated probabilistic locations. Although this sort of model has been used in the field of robot perception and planning before [24–26], its application to the calculation of the location matrices of dynamic obstacles, as far as we know, has not been presented in any previous works.

Given a sequence of observed positions $x_{0\cdots t}^{i}$, we wish to calculate the location matrix $m_{t}^{i}$ of object *i* at time *t*. The posterior probability for this parameter $p\left(m_{t}^{i}|x_{0\cdots t}^{i}\right)$ is given by Equation 1.

$$p\left(m_{t}^{i}|x_{0:t}^{i}\right)\;\propto \underset{\text{likelihood}}{\underset{︸}{p\left(x_{t}^{i}|m_{t}^{i}\right)}}\;\cdot \int \;\underset{\text{motion}\;\text{prior}}{\underset{︸}{p\left(m_{t}^{i}|m_{t-1}^{i}\right)}}\cdot \;\underset{\text{posterior}\;\text{at}\;t-1}{\underset{︸}{p\left(m_{t-1}^{i}|x_{0:t-1}^{i}\right)}}\;\text{d}m_{t-1}^{i}$$ (1)

#### Observation likelihood

The way to calculate the observation likelihood depends on the considered time step. All observations before the current time step *t*_*P*_ are determined by the measurement devices, and affected by whichever data post-processing they may require and inaccuracies they may suffer. These are usually modeled as Gaussian functions.

However, future observations cannot be obtained, and therefore no valid locations may be inferred from measurements after *t*_*P*_. In this case, the measured location has to be modeled as uniform over the entire working area (Equation 2).

$$\begin{array}{c} p\left(x_{t}^{i}|m_{t}^{i}\right)∼\left\{\begin{array}{cc} 𝒩\left(\mu_{t},\sigma^{2}\right) & \;\mathrm{if}\;0\leq t\leq t_{P}\\ 𝒰\left(W\right) & \;\mathrm{if}\;t_{P}<t\leq t_{T} \end{array}\right. \end{array}$$ (2)

#### Motion prior

The motion prior of each obstacle *i* is calculated as a convolution of its previous probable position. The movement of each object is modeled as a two-dimensional accelerated motion, such that Equation 3 is satisfied when *δ*_*t*_ is the time interval between two observations.

$$\begin{array}{cc} x_{t}^{i} & =x^{i}(t_{P}+t\cdot \;\delta_{t})\\ & =x^{i}(t_{P})+v^{i}(t_{P})\cdot \;(t\cdot \delta_{t})+\frac{1}{2}\cdot a^{i}\cdot \;{\left(t\cdot \delta_{t}\right)}^{2}\\ & =x_{0}^{i}+v_{0}^{i}\cdot \;(t\cdot \delta_{t})+\frac{1}{2}\cdot a^{i}\cdot {\left(t\cdot \;\delta_{t}\right)}^{2} \end{array}$$ (3)

The *x* ^*i*^ values are not deterministic positions, but essentially continuous probabilistic distributions; this is because they are inferred from previous measurements, that may be noisy or otherwise impossible to define using the expected motion model. As such, *v* ^*i*^ and *a* ^*i*^, which are in turn used to calculate new positions, will also be probabilistic.

In our work, the acceleration is modeled as a Gaussian function with mean $\mu_{a}^{i}$ and standard deviation $\sigma_{a}^{i}$, computed from the variation of the positions observed by the measurement devices. Both components of the two-dimensional movement are assumed independent and handled as different models.

In the case we present, the motion acceleration can also be modeled and rewritten as a function of position, time and velocity. Using Equation 3, we first define the variation between two positions as Equation 4.

$$\begin{array}{c} \Delta x_{t}^{i}=x_{t}^{i}-x_{t-1}^{i}=v_{0}^{i}\cdot \delta_{t}+a^{i}\cdot \left(t-\frac{1}{2}\right)\cdot \delta_{t}^{2} \end{array}$$ (4)

Rearranging this equation and connecting it to the probability distribution which *a* ^*i*^ follows, we obtain Equation 5.

$$\begin{array}{c} a^{i}=\frac{\Delta x_{t}^{i}-v_{0}^{i}\cdot \delta_{t}}{\left(t-\frac{1}{2}\right)\cdot \delta_{t}^{2}}∼𝒩\left(\mu_{a}^{i},{\left(\sigma_{a}^{i}\right)}^{2}\right) \end{array}$$ (5)

Applying a linear transformation to $a_{t}^{i}$, we obtain the probability model for the position variation, Equation 6. Notice how the variance increases quadratically over time.

$$\begin{array}{c} \Delta x_{t}^{i}∼𝒩\left(v_{0}^{i}\;\cdot \;\delta_{t}+\mu_{a}^{i}\;\cdot \;\delta_{t}^{2}\left(t-\frac{1}{2}\right),{\left(\sigma_{a}^{i}\right)}^{2}\;\cdot \delta_{t}^{4}{\left(t-\frac{1}{2}\right)}^{2}\right) \end{array}$$ (6)

A convolution mask is created so that it defines the probabilistic relative region of space where an object would travel, considering the calculated acceleration and accumulated velocity and position, according to Equation 6. Applied to the posterior location matrix of the corresponding object, the prior matrix at the following time step is obtained.

#### Integration procedure

This calculation however would consider both the obstacles and the robot as punctual. In order for our algorithm to work properly, the geometry of every element must be taken into account, as stated in [27].

To solve this flaw, the calculated location matrix for each object is integrated for a circular area of radius *r*_*i*_+*r*_*R*_, that is, its own radius plus the robot radius; for each navigable position *w* ∈ *W*, Equation 7 applies.

$$\begin{array}{c} p\left(m_{t}^{i}\left(w\right)\right)=\int_{{∥w-z∥}_{2}\leq r_{i}+r_{R}}p\left(m_{t}^{i}\left(z\right),x_{0:t}^{i}\right)dz. \end{array}$$ (7)

This way, the probability of a collision with a particular object is at least that of the closest point of said obstacle that the robot can impact.

This technique was tested along with those used in previous works, such as Minkowski sums [7, 8] and convolutions [28], and was found to be more efficient due to its greater computational simplicity: since our method depends exclusively on the radii of the involved objects, the integration boundaries remain constant during the entire execution, and can therefore be calculated beforehand.

#### Global collision probability matrix

Each position of the global matrix *M* is expected to inform about the combined collision probability of every obstacle, for each step *t* of the time window; therefore, a union must be applied. In this paper, we consider the probable locations of different objects to be independent, since they do not attempt to avoid each other.

The union rule for a high number of events is inefficient, since the intersections for all possible event combinations must be calculated (Equation 8).

$$\begin{array}{cc} p\left(\overset{N}{\underset{i=1}{⋃}}m_{t}^{i}\right) & =\sum_{i=1}^{N}p\left(m_{t}^{i}\right)-\sum_{i=1}^{N-1}\;\sum_{j=i+1}^{N}p\left(m_{t}^{i}\cap m_{t}^{j}\right)\\ & +\sum_{i=1}^{N-2}\;\sum_{j=i+1}^{N-1}\;\sum_{k=j+1}^{N}p\left(m_{t}^{i}\cap m_{t}^{j}\cap m_{t}^{k}\right)\cdots \end{array}$$ (8)

However, De Morgan’s laws simplify the calculation (Equation 9).

$$\begin{array}{c} M_{t}=p\left(\overset{N}{\underset{i=1}{⋃}}m_{t}^{i}\right)=p\left(\bar{{⋂}_{i=1}^{N}\bar{m_{t}^{i}}}\right)=1-\prod_{i=1}^{N}\left(1-p\left(m_{t}^{i}\right)\right). \end{array}$$ (9)

**Fig 1:**
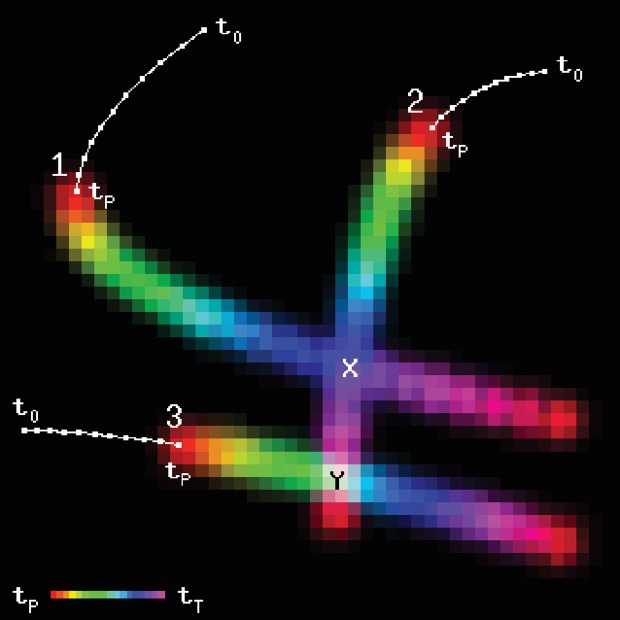
Collision matrix *M* for three moving obstacles. The observed trajectories are shown as white lines, and the predicted location is shown as a colored area. For visualization purposes, motion noise has been omitted.

Fig. 1 shows a global collision matrix *M* for three moving obstacles. Unless stated, in all figures the integration radii *r*_*i*_+*r*_*R*_ are equal to 1. The predicted trajectories are color coded, such that the hue value varies with time from *t*_*P*_ to *t*_*T*_. In order to simplify the graphics, the observed trajectories for *t* < *t*_*P*_ are not displayed from this point on.

Although obstacle collisions are not considered in this work, it can be observed that objects 1 and 2 would overlap on position X, since the color of their trajectory coincides at that point. Objects 2 and 3 however would not collide at position Y, since their colors differ.

### Optimal trajectory calculation

The result of the previous stage *M* is then used to generate a trajectory *u*(*t*) over *W* between two locations *x*_*start*_ and *x*_*goal*_, with a minimum collision probability and respecting the motion physics of the robotic device. For the sake of simplicity, a non-holonomic Dubins-like car [29] is simulated (Equation 10).

$$\begin{array}{c} \begin{array}{ccccc} \dot{x}=v\;\cos\theta & & \dot{y}=v\;\sin\theta & & \dot{\theta }\in \left[-\omega ,\omega \right] \end{array} \end{array}$$ (10)

However, the algorithm can be adapted to any kinematic model. By default, we use *v* = 1 and *ω* = *π*/4.

#### Time-dependent Dijkstra algorithm

To compute a safe route between two positions, we developed a simple time-dependent variation of the Dijkstra algorithm, shown in algorithm in Table 1. The motion physics defined for the robotic device are taken into account, both when checking which poses are accessible at each time step, and when calculating the cost of each movement. The accumulated collision cost of reaching position *y* from position *x* is given by Equation 11, where ‖*y*−*x*‖ is the movement cost and *M*_*t*_(*y*) is the collision probability of position *y*, at the moment when it is reached.

$$\begin{array}{c} C_{y}=C_{x}+∥y-x∥\times M_{t}\left(y\right) \end{array}$$ (11)

**Table 1:** Time-dependent Dijkstra algorithm

$C\;\left(x\right)\leftarrow \{\begin{array}{ll} 0 & \text{if}\;x=x_{start}\\ \infty & \text{if}\;x\in W-\left\{x_{start}\right\} \end{array}$	{Accumulated cost}
$t_{A}\;\left(x\right)\leftarrow \{\begin{array}{ll} 0 & \text{if}\;x=x_{start}\\ \infty & \text{if}\;x\in W-\left\{x_{start}\right\} \end{array}$	{Time of arrival}
*P* (*x*_*start*_) ← ∅	{Preceding location}
*V* ← {*x*_*start*_}	{List of recently visited locations}
**repeat**	
**extract** *x* from *V* such that $C\;\left(x\right)=\underset{v\in V}{\min}\left\{C\;\left(v\right)\right\}$	
**for** every *y* ∈ *W* accessible from *x* **do**	
*k* ← *C* (*x*) + ‖*y* − *x*‖ × *M*_*t*_*A*_(*x*)_ (*y*)	{Equation 11}
**if** *k* < *C* (*y*) **or**	
(*k* = *C* (*y*) **and** *t*_*A*_ (*x*) + *δ*_*t*_ < *t*_*A*_(*y*)) **then**	
*C* (*y*) ← *k*	
*P* (*y*) ← *x*	
*t*_*A*_(*y*) ← *t*_*A*_ (*x*) + *δ*_*t*_	
*V* ← *V* ∪ {*y*}	
**end if**	
**end for**	
**until** *V* = ∅	
*z* ← *x*_*goal*_	{Trajectory tracing}
**repeat**	
*u* (*t*_*A*_ (*z*)) ← *z*	
*z* ← *P* (*z*)	
**until** *z* = ∅	
**return** *u* (*t*)	

This method is arguably similar to the D* Lite algorithm [30], since the time of arrival at a cell does affect its cost, but our approach does not need to recalculate its route when finding an unexpected obstacle. This is so because all the steps of the evolution of the collision matrix are taken into account simultaneously during the computation of a route. However, in case of sequential execution, a precalculated route cannot be reused, since the collision matrix is completely reconfigured for each iteration of the algorithm.

**Fig 2:**
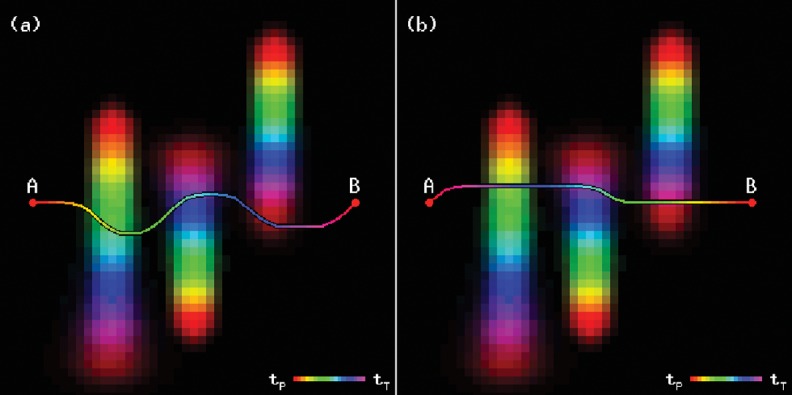
Trajectory through a crowded space, from point A to point B (a) and from point B to point A (b). Notice how the time step at which each location is reached does affect the trajectory.

Two examples of trajectories are shown in Fig. 2, where it can be observed that the vehicle avoids the paths of the objects only when they pose a risk.

The collision probability of the trajectory of the robot is finally computed as a union, such that the time at which each location is reached affects the result (Equation 12).

$$\begin{array}{c} p^{C}\;\left(u,M\right)=\overset{T_{u}}{\underset{t=1}{⋃}}M_{t}\left(u\left(t\right)\right) \end{array}$$ (12)

*T*_*u*_ is time span required for the robot to reach its destination (equivalent to *t*_*A*_ (*x*_*goal*_) in the algorithm in Table 1). The spatial length of the trajectory will be denoted as ‖*u*‖ (Equation 13).

$$\begin{array}{c} ∥u∥=\sum_{t=1}^{T_{u}}∥u\left(t\right)-u\left(t-1\right)∥ \end{array}$$ (13)

#### Minimum probability restriction

The presented solution may sometimes be excessively cautious and take unnecessary detours. By setting a fixed minimum value *ɛ*, such that we define a new collision probability matrix as shown in Equation 14, more reasonable trajectories can be produced. The default probability matrix can still be used by considering *ɛ* = 0.

$$\begin{array}{c} M_{t}^{\varepsilon }\left(x\right)=\left\{\begin{array}{cc} M_{t}\left(x\right) & \;\mathrm{if}\;M_{t}\left(x\right)>\varepsilon \\ \varepsilon & \;\mathrm{if}\;M_{t}\left(x\right)\leq \varepsilon \end{array}\right. \end{array}$$ (14)

This restriction is similar to the chance constraints method [6], which specifies to which areas of the working space the autonomous vehicle must restrict its movement, and which areas it must avoid. This technique was combined with the probabilistic collision method in [9] by quantizing the resulting probability matrix around a fixed value for each observed obstacle.

However, in our case, *ɛ* works as a lower bound that declares a certain area of the working space to be safe enough to travel. No upper bound is defined, as our algorithm is expected to always return the trajectory with the lowest collision probability; if we were to establish a certain maximum probability value and it still were lower than that of our trajectory, there simply would not be any valid solutions.

**Fig 3:**
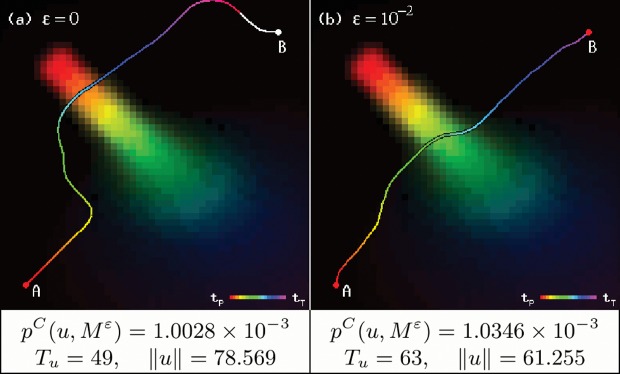
Effects of minimum probability restriction parameter *ɛ*, on a trajectory from point A to point B. The white segment in (a) is the section of the trajectory that exceeds time step *t*_*T*_, with *T* = 50.

Fig. 3 shows the results of using a *ɛ* value. In this example, a 0.01 minimum probability restriction produces a collision probability increase of only 3.17%, while the trajectory length is reduced by 22.037%, and the required time to reach the desired destination is $22.\bar{2}\%$ lower.

## Results

### Simulation results

**Fig 4:**
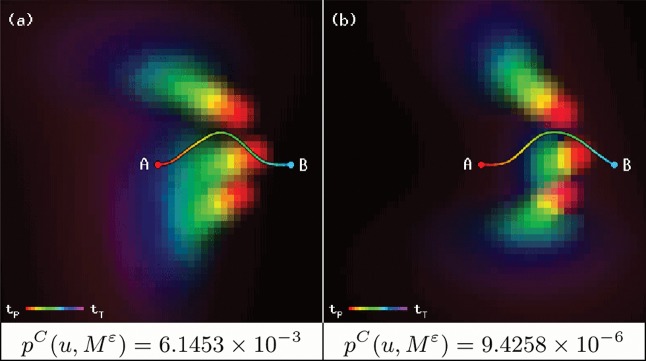
Trajectory calculation for the original examples using passive (a) and active (b) objects. We use *T* = 35 and *ɛ* = 10^−3^; in both cases, *T*_*u*_ = 21 and ‖*u*‖ = 24.142.

Our algorithm computes a trajectory with a minimum collision probability for different configurations of randomly moving objects. In order to compare the performance of our algorithm to previously available methods, we reproduce the examples given in [8] and force the robot to create a path through a probabilistically crowded area, as shown in Fig. 4, for both passive and active objects, along with the collision probabilities for the resulting trajectories. Since the original method did not take time into account, the results greatly differ: the robotic device is able to maneuver around the moving obstacles, instead of just choosing the optimal braking trajectory, and still shows a very low collision probability.

**Fig 5:**
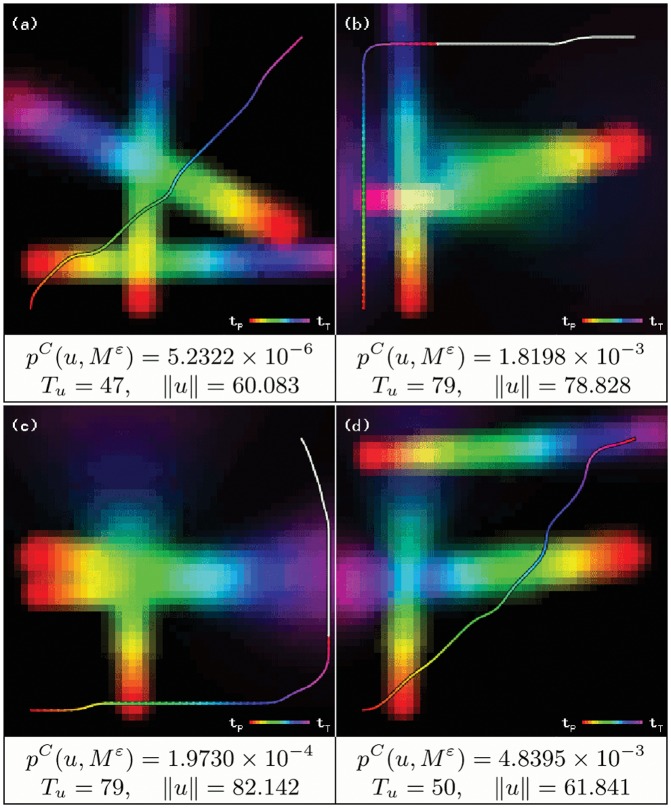
Examples of trajectories with minimum collision probability.

More complex configurations were automatically generated in order to thoroughly test our algorithm. Objects were programmed to move following initial randomized curve trajectories, such that they would always affect the calculations of the robot. Table 2 shows the average results for 100 executions of our simulation, along with the chosen parameters. Fig. 5 shows four examples and their results.

**Table 2:** Simulation results and parameter values.

Number of objects	*N* = 3
Size of time window	*T* = 50 time steps
Reduction parameter	*ɛ* = 10^−3^
Integration rad. object 1	*r*_*R*_ + *r*_1_ = 2
Integration rad. object 2	*r*_*R*_ + *r*_2_ = 1
Integration rad. object 3	*r*_*R*_ + *r*_3_ = 1
Average collision prob.	$\bar{p^{C}\;\left(u,M^{ɛ}\right)}=1.548\;\cdot \;10^{-3}$
Average trajectory time	$\bar{T_{u}}=53.96$ time steps
Average trajectory length	$\bar{\left\|u\right\|}=64.641$

Our algorithm produces very low collision probabilities for all cases, provided that the randomly obtained trajectories of the objects don’t pass through the initial and target positions, or completely fill the area in between them. In order to confirm the adaptability of our method, four additional kinematic models were programmed, and each one was tested over 100 instances of our simulation. As Table 3 shows, higher velocity values—both linear and angular—decrease the maneuverability of the robot, causing less efficient routes; however, the average collision probability remains similar to *ɛ* for all models.

**Table 3:** **Simulation results for different kinematic models**, given as variations of linear velocity *v* and angular velocity *ω*, for *ɛ* = 10^−2^ and *T* = 50 time steps.

*v*	1	1	2	2	3
*ω*	*π*/4	*π*/2	*π*/4	*π*/6	*π*/8
$\bar{p^{C}\;\left(u,M^{ɛ}\right)}$	1.1235⋅10^−2^	6.6372⋅10^−2^	1.3589⋅10^−2^	1.0794⋅10^−2^	1.1876⋅10^−2^
$\bar{T_{u}}$	64.40	81.32	44.88	28.32	21.10
$\bar{\left\|u\right\|}$	71.817 steps	80.320 steps	71.147 steps	60.759 steps	62.227 steps

#### Sequential execution

Although it provides valid trajectories, our algorithm is heavily based on probabilistic assumptions of the future behavior of perceived pedestrians, which are bound to change every time step. A more complex approach is therefore required in order to apply this method to real cases.

**Fig 6:**
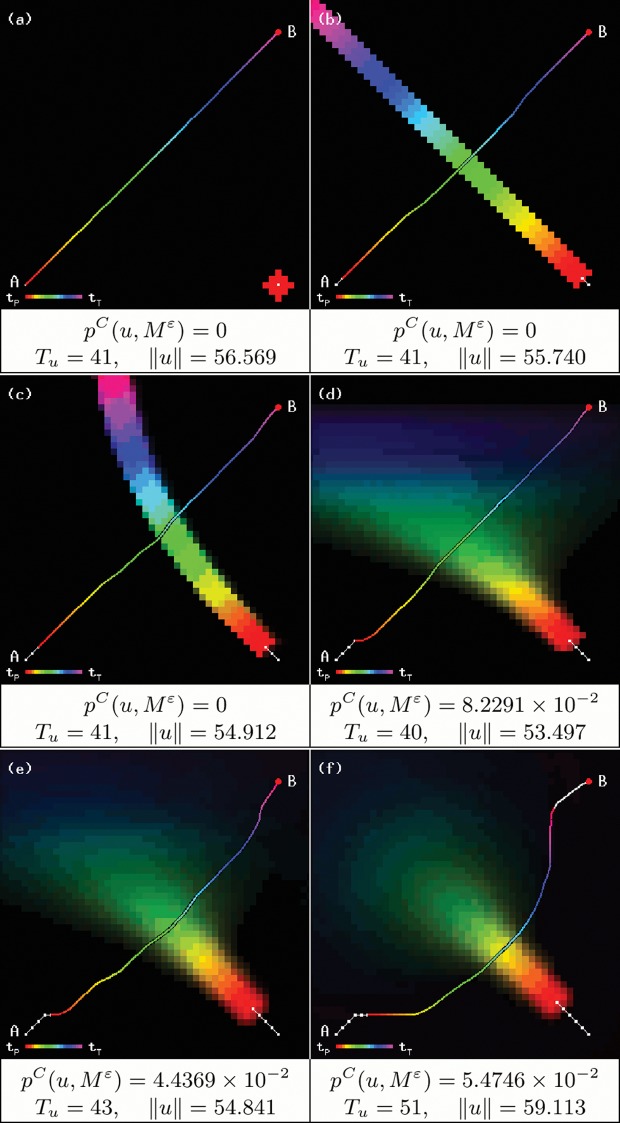
Example of sequential calculation of a trajectory from point A to point B with one obstacle. We use *T* = 45, *ɛ* = 10^−3^ and *r*_*R*_ + *r*_1_ = 2.

To do this, the trajectory is only followed for a certain amount of time, ideally the length of a time step, while the obstacles are observed and a new trajectory is processed. Fig. 6 shows an example of a sequential execution of the algorithm.

As can be seen, the expected future positions for the objects are more accurately calculated when more knowledge about their motion has been gathered. The limitations of the prediction procedure can also be observed: speed and acceleration are considered null or constant until enough observations have been collected.

Although this set of experiments assumes the robot moves at a constant speed, braking maneuvers can also be included in the motion model of the robot, so that more realistic trajectories are computed.

### Experimental results

**Fig 7:**
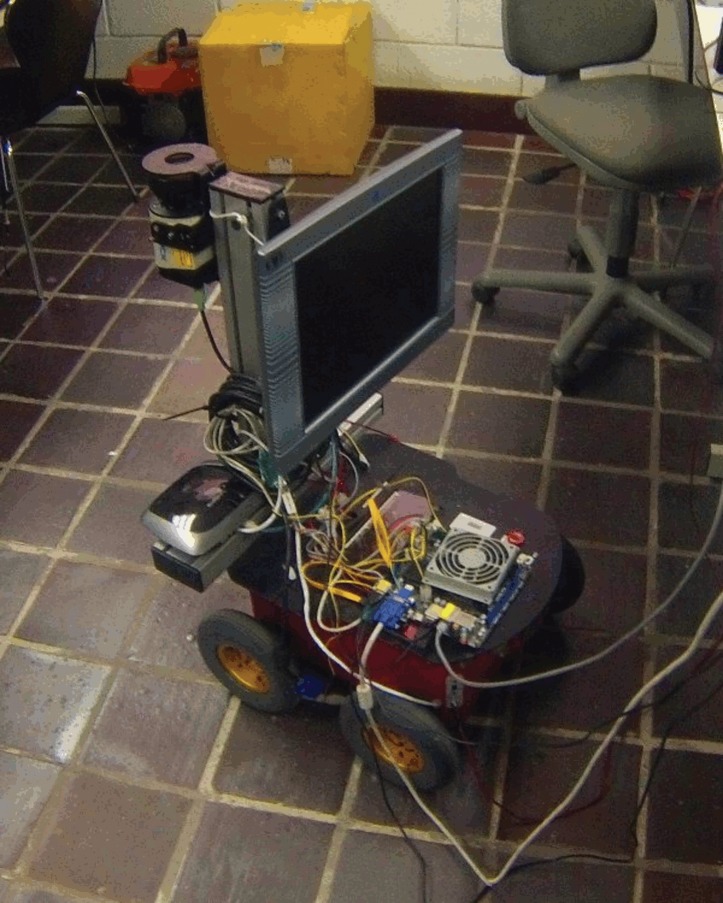
Robot used in our experiments.

After verifying its efficiency theoretically, our algorithm was installed on an Adept Pioneer 3-AT device (Fig. 7). This was equipped with a customized on-board computer, which included a Intel Core2 Quad processor with four 2.66 GHz cores, 4Gb RAM, and a 64-bit Ubuntu 12.04 operative system with ROS Hydro Medusa. Visual information was provided by a zenithal GoPro Hero3 camera installed on the ceiling of the test area.

**Fig 8:**
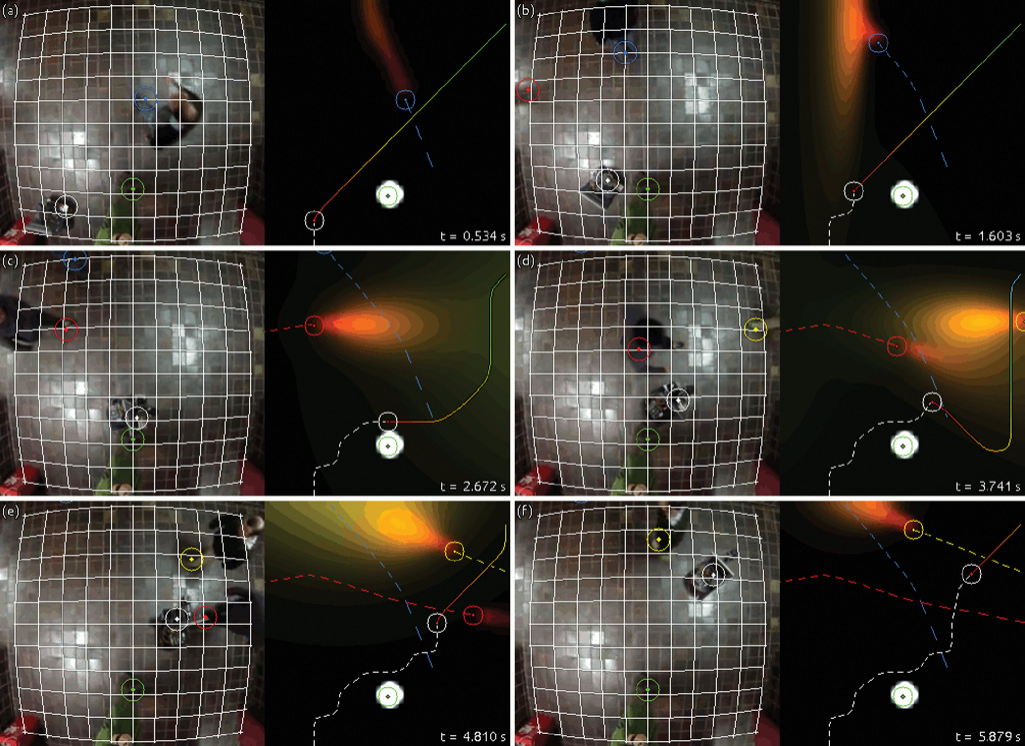
Example of sequential calculation of an optimal trajectory in a crowded environment. We use a workspace resolution of 50×50, a time window of *T* = 30 steps, and a minimum collision probability of *ɛ* = 0.1. The white dashed line represents the path that the robot has already followed, while the colored dashed lines show the observed movements of the obstacles.

Our robot was released in a 4.25*m*×4.25*m* area in a crowded corridor of our faculty, and commanded to reach different target positions, following trajectories with minimum collision probability, which were computed sequentially as explained in the previous subsection. Fig. 8 shows an example of the calculation of one of these trajectories. Pedestrians were given an integration radius of one meter.

We define two new variables: *δ*_*u*_, which represents the relative increment between the traveled distance and the length of the minimum path between the starting and goal poses (Equation 15), and the processing time for a complete iteration (calculation of both a probabilistic collision matrix and an optimal trajectory), which we named *τ*.

$$\begin{array}{c} \delta_{u}=\frac{∥x_{goal}-x_{start}∥-∥u∥}{∥x_{goal}-x_{start}∥} \end{array}$$ (15)

In order to test the performance of our algorithm in real time, we measured the average values of these parameters, along with the average collision probability of the final trajectory *p* ^*C*^ (*u*,*M* ^*ɛ*^), for 100 different configurations of workspace resolution, time window length *T* and minimum collision value *ɛ*.

The results are shown in Tables 4, 5 and 6. Notice that *p* ^*C*^ (*u*,*M* ^*ɛ*^) and *δ*_*u*_ are not calculated over the output of each iteration, but over the final trajectory of the robot resulting from the whole execution.

**Table 4:** Average processing time *τ*.

*T*	*ɛ*	Workspace resolution (*n*×*n*)				
		20	30	50	80	120
3	0	2.308	6.667	25.49	96.75	306.0
	0.05	1.818	7.273	27.88	104.2	332.9
	0.1	2.727	6.875	27.69	103.0	331.4
	0.5	2.778	5.926	25.56	105.7	335.7
5	0	2.500	6.750	27.64	103.2	320.3
	0.05	2.609	7.223	29.23	107.5	340.9
	0.1	2.727	7.273	27.84	105.4	341.4
	0.5	2.222	6.666	26.00	105.8	346.0
10	0	2.424	6.136	28.15	97.52	350.0
	0.05	2.692	7.179	30.16	110.7	355.5
	0.1	2.308	7.576	28.70	112.8	360.7
	0.5	2.222	6.296	26.44	110.7	361.2
20	0	2.188	6.600	28.06	116.5	398.8
	0.05	2.692	7.180	30.00	119.9	390.8
	0.1	2.308	7.105	30.67	117.9	390.6
	0.5	2.692	6.667	27.33	115.4	389.2
30	0	2.188	6.531	27.38	115.4	415.2
	0.05	2.692	7.436	29.39	124.9	413.1
	0.1	2.308	7.368	30.22	120.8	411.5
	0.5	2.778	5.926	27.11	116.9	409.3

Average processing time *τ* of one iteration (in ms), for each configuration of workspace resolution (in cells), size of time window *T* (in steps), and minimum probability value *ɛ*.

**Table 5:** Average length increment *δ*_*u*_.

*T*	*ɛ*	Workspace resolution (*n*×*n*)				
		20	30	50	80	120
3	0	26.88	22.80	8.599	7.432	5.701
	0.05	13.34	12.89	9.583	8.045	4.886
	0.1	13.33	11.24	9.583	7.432	3.665
	0.5	3.339	2.985	2.697	2.534	2.443
5	0	31.88	24.46	12.53	8.045	6.108
	0.05	15.84	17.85	9.583	8.044	4.072
	0.1	13.34	12.90	8.599	7.432	2.850
	0.5	3.340	2.985	2.671	2.533	2.443
10	0	54.98	31.06	22.15	232.4	7.737
	0.05	26.87	22.80	19.42	9.269	6.515
	0.1	23.34	12.89	3.681	2.534	2.442
	0.5	3.330	2.984	2.697	2.533	2.443
20	0	52.48	54.99	29.26	22.13	15.48
	0.05	26.88	22.80	21.39	8.045	2.442
	0.1	26.87	21.15	2.697	2.533	2.443
	0.5	26.88	2.984	2.696	2.534	2.442
30	0	52.48	53.34	46.87	29.37	22.16
	0.05	26.88	22.80	23.36	11.72	2.442
	0.1	26.87	21.15	2.697	2.534	2.443
	0.5	3.339	2.985	2.696	2.533	2.443

Average length increment *δ*_*u*_ of the resulting trajectory (in %), for each configuration of workspace resolution (in cells), size of time window *T* (in steps), and minimum probability value *ɛ*.

**Table 6:** Average collision probability *p* ^*C*^ (*u*,*M* ^*ɛ*^).

*T*	*ɛ*	Workspace resolution (*n*×*n*)				
		20	30	50	80	120
3	0	10^−3^	10^−3^	10^−9^	10^−9^	10^−9^
	0.05	0.127	0.094	0.050	0.050	0.052
	0.1	0.173	0.100	0.099	0.099	0.100
	0.5	0.545	0.528	0.510	0.500	0.500
5	0	10^−6^	10^−7^	10^−9^	0.033	10^−9^
	0.05	0.122	0.050	0.050	0.050	0.503
	0.1	0.187	0.115	0.101	0.099	0.100
	0.5	0.546	0.530	0.509	0.500	0.500
10	0	10^−6^	10^−6^	10^−7^	10^−8^	10^−9^
	0.05	0.050	0.050	0.050	0.050	0.504
	0.1	0.100	0.114	0.105	0.099	0.100
	0.5	0.543	0.531	0.509	0.500	0.500
20	0	10^−6^	10^−6^	10^−7^	10^−9^	10^−4^
	0.05	0.050	0.050	0.050	0.050	0.050
	0.1	0.100	0.103	0.108	0.099	0.100
	0.5	0.542	0.530	0.509	0.500	0.500
30	0	10^−6^	10^−6^	10^−7^	10^−9^	10^−4^
	0.05	0.050	0.050	0.050	0.050	0.050
	0.1	0.100	0.103	0.108	0.099	0.100
	0.5	0.543	0.530	0.508	0.500	0.500

Average collision probability *p* ^*C*^ (*u*,*M* ^*ɛ*^) of the resulting trajectory, for each configuration of workspace resolution (in cells), size of time window *T* (in steps), and minimum probability value *ɛ*.

## Discussion

As we expected, experimental data shows that the processing time of the algorithm depends almost exclusively on the workspace resolution. Since every iteration of the procedure must also include the stages of data extraction from the visual media, as well as command delivery to the motion system, this parameter must be finely tuned in order to obtain precise enough trajectories in low enough time spans.

Increments of this value also cause shorter trajectories. This happens because a higher number of cells allows the algorithm to consider more accurate movements over the working area. However, the time cost of this precision can cause the robot to skip steps and calculate faulty trajectories. In our experiments, a workspace resolution of 50×50 cells resulted in a good enough avoidance behavior.

Higher values of *ɛ* result in shorter trajectories, along with higher collision probabilities—experimentally, we observed that the robot behaved more recklessly and moved closer to the obstacles. However, *ɛ* = 0 could cause extremely inefficient routes, with long detours and braking maneuvers. A value of *ɛ* = 0.1 was found to give good enough results for our particular case. Notice how, as our calculations are made a posteriori over the final result of the algorithm, the average collision probability of each trajectory closely resembles *ɛ*.

Finally, the size of the time window *T* was not observed to affect the processing time significantly, as the algorithm in Table 1 explores the whole workspace regardless of the available collision data. However, higher values were found to produce shorter trajectories and lower collision probabilities, since the accuracy of the method increases. Therefore, it is advised to make this value equal to the expected time span required for the robot to reach its goal.
